# Impact of DNA methylation on trophoblast function

**DOI:** 10.1186/1868-7083-3-7

**Published:** 2011-11-01

**Authors:** L Serman, D Dodig

**Affiliations:** 1 Department of Biology, School of Medicine, University of Zagreb, Zagreb, Croatia; 2 County of Krapina - Zagorje Medical Centre, Office of Family Medicine, Krapina, Croatia

## Abstract

The influence of epigenetics is evident in many fields of medicine today. This is also true in placentology, where versatile epigenetic mechanisms that regulate expression of genes have shown to have important influence on trophoblast implantation and placentation. Such gene regulation can be established in different ways and on different molecular levels, the most common being the DNA methylation. DNA methylation has been shown today as an important predictive component in assessing clinical prognosis of certain malignant tumors; in addition, it opens up new possibilities for non-invasive prenatal diagnosis utilizing cell-free fetal DNA methods. By using a well known demethylating agent 5-azacytidine in pregnant rat model, we have been able to change gene expression and, consequently, the processes of trophoblast differentiation and placental development. In this review, we describe how changes in gene methylation effect trophoblast development and placentation and offer our perspective on use of trophoblast epigenetic research for better understanding of not only placenta development but cancer cell growth and invasion as well.

## 1. Introduction

After completing the human genome sequencing in 2000, the world of science thought it overcame the final barrier to understanding the mystery of life. What it stumbled upon, however, really has been just another puzzle and a new riddle to solve. Knowing does not necessarily imply easy understanding.

Genetics and cell science have revealed in detail what cells in our body are made of, but have not yet succeeded in explaining the diversity in morphology and function that derives from the same genetic material. Explaining the mechanisms that regulate temporally and spatially dependent expression of selected genes in guiding cell differentiation and function, for processes as divergent as embryo development and schizophrenia, is the ground work of epigenetics [1]. The fact that extrinsic environmental stressors can influence chromatin structure and lead to cell phenotype change, which could then be passed on to the next generation without disturbing the DNA sequence, dramatically challenges the basic Mendelian postulates [2].

Most of the current research in epigenetics focuses on regulation of gene transcription by changes in chromatin form and structure, via acquisition of new covalent or non-covalent bonds, through DNA methylation or histone proteins acetylation, methylation and phosphorylation [3,4]. Methylation of the DNA CpG base pairs, located predominantly in the promoter regions of the genes, disables binding of transcription factors, thus "silencing" the gene expression, whereas modifications to histone proteins and nucleosomes influence chromatin form by changing the charge of histone tails and making the DNA physically either more or less accessible to transcription factors [5,6].

Though the epigenetic mechanisms are themselves quite simple, easy to explain and reproduce, the complex network of interactions which they induce in order to produce a unique pattern of gene expression, is immensely complex and poses a serious challenge for current and future research in the field of epigenetics [7,8]. Despite the complexity, epigenetic research offers insights that will likely result in better explaining of diverse entities in human physiology and pathology that are currently insufficiently understood - entities such as cell migration and differentiation (e.g. during implantation, placentation, embryogenesis and organogenesis), pathology of the placenta (including pre-eclampsia, eclampsia, HELLP syndrome and intrauterine growth restriction syndrome (IUGR)), carcinogenesis, brain plasticity and effects of environmental factors on growth, development and health, among many others.

In this regard, certain steps have already been made toward the practical application of this knowledge in modern medicine. Thus, methylation status of the APC1A tumor suppressor gene promoter has been suggested as a novel marker of an unfavourable outcome in cervical cancer, i.e. prognosis is worse with an increase in promoter methylation [9]. The relationship between APC1A methylation and cervical cancer clinical outcome may be explained by known functions of the APC1A in cell adhesion and β-catenin inactivation. This has been confirmed in recent *in vitro *studies, where addition of hydralazine to the cervical cancer cell lines (including HeLa), resulted in demethylation of the APC promoter and consequent cell growth inhibition and disruption of the β-catenin expression [10].

Gene inactivation by promoter hypermethylation has been also discovered within the large group of Ras effectors with tumor suppressor activity (although Ras family of small GTP-linked proteins may act to promote tumorigenesis as well, depending on the downstream activated molecules). For example, NORE1A (RASSF5) protein is a family member with the typical Ras characteristic domain. Its role as a tumor suppressor is accomplished through induction of apoptosis and delay in cell cycle progression in various cancer cell lines. Thus, it is frequently silenced in human tumors, most commonly due to methylation of its promoter [11]. Related protein, RASSF1A (Ras association domain-containing protein 1), can be inactivated in a similar fashion. NORE1A and RASSF1A form heterodimers and activate proapoptotic signals. RASSF1A gene promoter is one of the most commonly hypermethylated tumor suppressor genes found in human tumors, and its methylation is in direct correlation with its gene expression level [12].

## 2. Rodent placenta as a model for epigenetics of human placental development

Since mouse and rat placentas are morphologically and genetically similar to human placenta, the results gathered in epigenetic research in rodents can be applied to humans [13]. Most important functional features of both mouse/rat and human placentas are the invasion of endometrium by trophoblast cells and the nutrient and gas exchanges in placental labyrinth layer (in rodents; analogous to the chorionic villi in humans), which is covered by syncytiotrophoblast laying in direct contact with maternal blood [14].

Furthermore, several genes, such as Igf2, H19, Mash2, Hand1, Gcm1 and Met, are found to be important during both rodent and human placental development, indicating common molecular mechanisms [15]. However, their distinct functions in humans is yet to be investigated further [14].

Numerous human analogues of the mouse genes have been identified, such as an analogue of the mouse Mash2 gene, which is important for the spongiotrophoblast development. Expression of Mash2 is regulated by genomic imprinting, since the identical phenotype is seen in mice with mutations in both alleles (homozygous mice) as well as in those heterozygous mice with maternally inherited mutant allele, whereas heterozygous mice with paternally inherited mutant allele remain unaffected. Mash2 human analogue, HASH2 (Human Achaete-Scute Homologue 2), is expressed in extravillous trophoblast; since its presence is lacking in androgenic hydatidiform moles, its expression is thought to be imprinted as well [16,17]. Another murine gene, Gcm-1 (glial cells missing homologue 1), blocks mitosis and is essential for labyrinth development and syncytiotrophoblast differentiation; its human analogue, GCM1, displays similar expression pattern. Inhibition of the GCM1 in human placental cell lines prevents exravillous trophoblast differentiation, whereas addition of forskoline, an inducer of GCM1 expression, leads to an increased syncytialization and decreased trophoblast proliferation. Taken together, alterations in GCM1 gene expression may explain certain aspects of pre-eclampsia and IUGR-associated clinical pictures [18].

Since cells making up placenta display unique methylation patterns compared to somatic cells, the placental development has been recognized as one of the best model for studying epigenetic mechanisms [19,20]. During the complex processes of implantation and placentation, coordinated epigenetic regulation ensures that the sequence of gene expression in cells is accurate and timely. DNA methylation is thought to be the main epigenetic mechanism operating during placental development; any disruption in placental methylation pattern leads to aberrant function of the placenta [21]. The trophoblast cells of the placenta have the lowest 5-methylcytosine content of all human cells (except germ cells), comparable to hypomethylated levels in cancer cells and have a unique set of imprinted genes, making them a rich source for research on the impact of DNA methylation on physiological and pathological tissue development [22-28].

Although rodent placenta represents a suitable model for epigenetic studies, and as such, a model for placentation in humans, there are however, certain differences in epigenetics between rodent and human placentation that should be kept in mind. In mice, imprinted genes, of which there are many in the placenta, are largely maternally expressed and paternally repressed [29]. In humans, however, gene imprinting mechanism may differ, as some imprinted genes are maternally (e.g. APC, WIF1) and others paternally (SFRP2, RASSF1A) expressed [30]. Further, the imprinting of the ESX1L gene is indispensable for placental development in mice, whereas that same homeobox gene is found not to be imprinted in humans, during the last trimester of pregnancy [31]. Its expression is significantly lower in pregnancies associated with IUGR, suggesting that some idiopathic fetal growth restrictions are associated with placental dysfunction [32]. Also, expression of RASSF1A in human placenta is highest in stroma and lowest in cytotrophoblast, where it is extensively methylated. Hypermenthylated Rassf1 is found in the placentas of Rhesus monkeys as well. In the mouse placenta, however, that same gene is not methylated; additionally, mice with knocked out Rassf1 are fertile and display no reproductive disturbances, which shows that this imprinted gene has not been evolutionary conserved [33].

The use of experimental animal models inevitably raises questions about their value for understanding human physiology and disease. However, animal models are unavoidable, and as long as one is careful in interpreting the results obtained on them, and until those results are confirmed on human tissues, they are of great scientific value. The benefit to the use of experimental animals is also the ability to construct null-mutants for the particular gene of interest, with the aim of discovering its function in the placenta.

In addition to rodent vs. human, anatomical and physiological differences between mice and rats have to be appreciated as well. For example, trophoblast invasion into uterine epithelium is shallow in mice whereas it is deeper in rats (mesometral triangle) and as such more similar to humans [34].

The discovery of the cell-free fetal DNA (cffDNA), which amounts to about 5% in the mother's circulating blood, and is being used for fetal RhD status and sex determination, opens up new possibilities for non-invasive diagnostics [35]. In a light of recent findings, the above mentioned RASSF1A is being considered as the potential universal marker to aid detection of fetal DNA. Namely, since cffDNA is found in anembryonal pregnancies as well, it is thought that its source is syncytiotrophoblast [36]. The RASSF1A promoter is hypermethylated in the placenta and hypomethylated in the mother's blood - this pattern of methylation makes it possible to discriminate between the placental and mother's RASSF1A [37]. In addition, RASSF1A methylation level can discriminate between normal pregnancy and pre-eclampsia: whereas there are no differences in methylation between placentas, plasma RASSF1A is about four times more methylated in the pre-eclampsia patients than in women with normal pregnancies. Y-chromosome detection has been used as a marker of elevation of fetal DNA before the onset of pre-eclampsia, but methods based on RASSF1A methylation level now make non-invasive diagnosis of pre-eclampsia possible as well [38].

Finally, in addition to mere presence or absence of some tissue-specific factors, recent findings suggest that for certain genes, such as Cdx2 and Oct4, their amount relative to other factors is important as well. Such ratio of factors leads to establishment of a specific cell types, in this case trophoblast and embryoblasts cells inside the blastocyst [39].

We cannot close this chapter without addressing trophoblast stem cells, which so far have been successfully isolated from the mouse. As for the rat, a recently published report describes for the first time isolation and culture of cells that express markers specific for rat trophoblast stem cells (in the presence of FGF4) and their ability to differentiate into trophoblast giant cells (when FGF4 is removed from the medium) [40]. In human cells, overexpression of a single transcription factor, Oct4, in trophoblast cells is sufficient for their reprogramming into pluripotent cells (OiPS, Oct4-induced pluripotent cells). OiPS bear the epigenetic characteristics of embryonal stem cells (including reactivation of the inactivated X-chromosome, Oct4 and Nanog gene promoters hypomethylation and Elf5 gene promoter methylation) and are capable of differentiation into cells of all three embryonal layers [41].

## 3. Our epigenetic point of view on trophoblast diferentiation and development of placenta

The DNA methylation can be changed by exposure of cells to a well known demethylating agent 5-azacytidine (5azaC), which incorporates into both DNA and RNA molecules [42]. In DNA, it inhibits methylation by trapping DNA methyltransferase (Dnmt), resulting in its deactivation [43]. The 5azaC is a well known antineoplastic citidine analogue that, due to its inhibition of Dnmt and consequent promoter hypomethylation of transcriptionally silenced genes and cytotoxicity, is being used in therapies of myelogenous leukaemia and myelodysplastic syndrome [44]. For example, it has been shown that in the presence of 5azaC, the inhibitory effect of glucose on the expression of hGH-V (human placental growth hormone) is reduced, as 5azaC hypomethylates hGH-V (normally, high glucose levels lower the endogenous hGH-V mRNA) [45]. The potential use of 5azaC in therapy of solid tumors is currently being tested in clinical trials.

DNA methylation is considered as a barrier mechanism, one that in, e.g., globin genes ensures the balance between genes being accessible or not to the transcription machinery. Namely, in the absence of methylation, other accessory mechanisms of repression are not sufficient for histone deacetylation and consequent gene inactivation characteristic for non-erythroid cells. Thus, in these cells, all globin genes are in the form of heterochromatine, packed in the conformation that is insensitive to DNAse I and replicates late. However, simultaneous exposure to 5azaC and biturates is capable of stimulating the gamma-globin expression in patients with beta-hemoglobinopathies [46]. Over 30 genes with hypermethylated promoters in human lung cancers have been described in the last two decades. Cigarette smoke condensate hypermethylates promoters of the genes involved in epithelial-mesenchymal transition; thus, when human bronchial epithelial cell lines, exposed to cigarette smoke concentrate, are treated with 5azaC and trichostatin A (histone deacetylase inhibitor), a third of the silenced genes become reactivated [47].

The assumption that the effects of 5azaC are mediated solely through changes in DNA methylation, however, is not correct. Numerous studies have emerged in recent years demonstrating that the effects of 5azaC are cell and gene specific. For example Komashko and Farnham showed that (1) 5-azacytidine treatment results in large changes in gene regulation with distinct functional categories of genes showing increased and decreased levels; (2) most genes that show altered expression are not regulated by promoters that display DNA methylation prior to the treatment; (3) certain gene classes switch their repression mark upon treatment with 5-azacytidine; and (4) most changes in gene expression are not due to relief of repression mediated by DNA or histone methylation [48].

The prevalent outcome of DNA demethylation is expression of previously silenced genes, made possible by enabling attachment of transcription factors to the genes, with consequent gene transcription and protein synthesis up-regulation [49]. In our laboratory, after administering 5azaC to pregnant rats on different gestation days, we concluded that expression of certain genes is enhanced or even activated, in agreement with other investigators. Specifically, when looking at the distribution of placental glycoproteins, we detected some in the experimental group (with 5azaC) that were not present in the control (without 5azaC) rats [50].

On the other hand, expression of some genes is suppressed, rather than activated, upon demethylation. The well known example is the regulation of expression of closely linked and imprinted genes H19 (transcribed from maternally inherited allele) and Igf2 (transcribed from paternally inherited allele) [51]. The Igf2 was the first discovered imprinted gene - its targeted mutation leads to dwarfism in heterozygous individuals, but only when paternal (and not maternal) gene is affected. Growth reduction in newborns is identical in heterozygous individuals with mutated paternal gene and in recessive homozygous individuals, suggesting that the activity of the Igf2 gene is influenced only by the father [52]. Imprinted genes tend to cluster, which makes it easier to control their expression. This control is the responsibility of the cis-regulatory sites, such as the "imprinting control regions" (ICR) or "differentially methylated domains" (DMD), their characteristic being that they are always methylated on one of the two parental alleles [53,54]. If deletions in these sites occur, imprinting is lost and both alleles become expressed [55]. One of the best studied ICRs is the one situated within the H19/Igf2 imprinted gene complex, where activity of these two genes is dependent on the methylation status of the control region situated between them [56,51]. Specifically, on paternal chromosome, ICR is methylated, which makes H19 gene inactive and allows expression of Igf2 [57]. On maternal chromosome, ICR is not methylated and behaves as an "isolator", i.e. regulatory element that influences gene expression, onto which CTCF transcriptional regulatory protein can bind (it recognizes CCCTC sequence, CCCTC-binding factor (zinc finger protein)). As a result of CTCF binding, Igf2 promoter enhancer activity is inhibited and, consequently, Igf2 becomes inactive, whereas H19 gene is now active [58]. Thus, methylation can activate gene activity in an indirect manner, via blockage of an isolator [59]. The regulation of H19 gene expression is direct, since its methylation introduces inactive, i.e. imprinted site, and thus methylation directly influences its promoter activity [60].

Furthermore, in our research of epigenetic control of rat placentation, we obtained other interesting results concerning the proliferating capacity of trophoblast. We observed that 5azaC (i.e. hypomethylation) increases the expression of PCNA (proliferating cell nuclear antigen, a marker of cell proliferation) in trophoblast cells of rat placenta, with peak proliferation present when 5azaC is administered during the periimplantation period, from 4th to 6th day of gestation [50].

Recently, epigenetic research has gone even further by showing that methylation of the CRE (cAMP response element) sequence (TGACGTCA) enhances DNA binding of the C/EBPa transcription factor (CCAAT/enhancer-binding protein alpha), a protein critical for activation of differentiation of various cell types [61]. Transfection assays show that C/EBPa activates CRE sequence only when it is methylated, whereas experimental demethylation following 5azaC treatment diminishes C/EBPa binding and activation of the promoters during differentiation [61]. The lack of some glycoproteins in our experimental rat groups after 5azaC administration could probably be explained in similar fashion.

Moreover, research on the gene expression controlling effects of 5azaC is having a significant contribution for the understanding of tumor cell biology and mechanisms of oncogenesis as well. For example, in our laboratory, we have developed a model system for analysis of behaviour of rat trophoblast cells, which resemble tumor cells in many of their functional characteristics. In this system, trophoblast cells are transplanted under the rat kidney capsule and, mimicking cancer cell lines, allowed to grow and expand [62]. Then, post-implantation, cells are exposed to demethylating activity of 5azaC for various lengths of time and their growth and differentiation *in vivo*, as well as expression of a range of differentiation and cancer-associated genes in cells *ex vivo*, are being determined.

## 4. Conclusion

The DNA methylation, as one of the most important epigenetic mechanisms of gene expression control, offers a more complete insight into how this control is achieved in cells. It enables us to understand how a certain pattern of gene expression is accomplished in healthy tissues and in diseased cells of various pathologies. Trophoblast cells are the principal cells of normal embryonal implantation and placentation, as well as many pathologic conditions of pregnancy, such as eclampsia (where invasion of trophoblast cells is thought to be too shallow) or placenta accreta or preccreta (where invasion of cells is too deep). Epigenetic mechanisms can help explain the function of trophoblast cells in these conditions. By exposing these cells to the DNA demethylating agent 5azaC, we have been able to artificially demethylate or hypomethylate their DNA and, thus, interfere with expression of the genes crucial for the functioning of the trophoblast cells during embryogenesis. Such manipulated trophoblast cells have been employed in our unique rat animal model, a combination of in *vitro, in vivo *and *ex vivo *cell growth. We have used this model to investigate not only placental development, but mechanisms of tumor cell growth as well, as trophoblast and malignant cells share many of their functional characteristics, the most important being tissue invasion. Thus, determination of the sequence of molecular events that lead to timely and tightly controlled blockage of invasion of trophoblast cells during implantation could be directly translated into explaining why such mechanisms have been lost in cancer cells during metastasis.

## Conflict of interests

The authors declare that they have no competing interests.

## Authors' contributions

LS designed the article and participated in writing all sections of the manuscript. DD drafted the manuscript and participated in writing the first, second and third part of the manuscript. Both authors read and approved the final manuscript.
